# Survival Determinants in Glioblastoma: An Insight into Biopsy-Only Patient Outcomes

**DOI:** 10.3390/biomedicines12102327

**Published:** 2024-10-13

**Authors:** João Meira Gonçalves, Francisca Ferreira, Bruno Carvalho, Patrícia Polónia, Paulo Linhares

**Affiliations:** 1 Neurosurgery Department, Centro Hospitalar Universitário São João, Alameda Professor Hernâni Monteiro, 4200-319 Oporto, Portugal; 2 Faculty of Medicine, University of Porto, Alameda Professor Hernâni Monteiro, 4200-319 Porto, Portugal; 3 Neurology Department, Centro Hospitalar Universitário São João, Alameda Professor Hernâni Monteiro, 4200-319 Oporto, Portugal

**Keywords:** glioblastoma, biopsy, survival outcomes, adjuvant therapy

## Abstract

**Background:** Glioblastoma is a challenge in neuro-oncology, with survival significantly influenced mainly by the extent of resection and molecular markers. Despite advancements, the prognosis for IDH-wildtype glioblastoma remains poor, particularly when surgical resection is not possible. However, some patients exhibit unexpectedly extended survival despite the extent of resection. This study aims to analyze the determinants that contribute to these atypical survival rates among glioblastoma patients who have had solely biopsy procedures. **Methods:** We conducted a retrospective analysis of patients diagnosed with IDH-wildtype glioblastomas at our institution from 2017 to 2021, who underwent biopsy only. This study focused on evaluating the impact of demographic characteristics, clinical features, molecular markers, and treatment modalities on survival outcomes (overall survival (OS) and progression-free survival (PFS)). Statistical analyses included survival analysis and logistic regression for evaluating associations between OS and pre-operative characteristics and post-operative treatments. **Results:** The cohort included 99 patients, with a median age at diagnosis of 65.5 years. Median OS and PFS were 6.0 and 3.6 months, respectively. The multivariate analysis revealed that higher Karnofsky Performance Status (KPS) scores before biopsy, no contrast uptake on imaging, and any adjuvant therapy, particularly the use of bevacizumab, were independently associated to increased OS (HR = 0.97, *p* = 0.009. HR = 0.7, *p* = 0.015; HR = 0.27, *p* = 0.002, respectively). Out of 99 patients, 77.8% survived past the 3-month threshold, with 87.0% of this receiving adjuvant treatment. Only 8% of patients survived past 24 months, and in this group of patients, *MGMT* methylation was observed in just 25% of cases. Kaplan–Meier analysis indicated a better prognosis with any type of adjuvant therapy across all patients, particularly so in those with KPS ≥ 70. Age did not significantly affect survival outcomes (OR = 1.00, *p* = 0.835). **Conclusion:** Our findings reveal that any adjuvant treatment (whether chemotherapy and radiotherapy combined, chemotherapy alone, or bevacizumab), no contrast uptake on imaging, and higher pre-operative KPS are key determinants of survival in IDH-wildtype glioblastoma and should therefore be considered when deciding whether to perform a biopsy.

## 1. Introduction

Based on the 2021 WHO classification, the revised definition of glioblastoma emphasizes the molecular features over traditional histopathological criteria. Glioblastoma is now exclusively defined as an IDH-wildtype astrocytic tumor [1]. This shift toward molecular diagnostics is important, as it reduces dependance on morphologic features, which previously showed inconsistency in diagnosis. Importantly, even in cases where the tumor may display lower histological grades (grade 2 or 3), the presence of specific molecular markers, such as *TERT* promoter mutations and EGFR amplification, supports the classification as glioblastoma [2,3].

Within the clinical spectrum of gliomas, the prognostic panorama reveals a distinct divergence between grades. For individuals diagnosed with low-grade gliomas (e.g., IDH mutant), the expected survival spans an average of 7 years. In contrast, for those with high-grade gliomas, particularly glioblastoma with IDH-wildtype (IDH-WT), survival is significantly shorter, typically not exceeding 14 to 16 months, despite the employment of gold standard treatments such as gross total resection, radiation, and chemotherapy (Stupp protocol) [4].

The superiority of complete surgical resection over biopsy in the treatment of gliomas is a well-established principle in neuro-oncology. Surgical intervention, particularly gross total resection (GTR), is central to this strategy, offering significant benefits over biopsy alone. This approach directly reduces the volume of cancerous cells, potentially delaying the progression of the disease. Secondly, by decreasing the tumor burden, GTR enhances the effectiveness of adjuvant therapies, such as chemotherapy and radiotherapy [5,6,7].

The choice between biopsy and surgery for glioblastoma varies according to the tumor location, patient health, and the intervention’s goal. Biopsy is preferred for inaccessible tumors, high-surgical-risk patients, or when diagnostic clarity is needed. Multifocal or diffusely infiltrating tumors are another example. Decisions should be patient-specific, weighing benefits against risks [8,9].

Conversely, a growing body of clinical evidence reveals a subset of patients who, despite only undergoing biopsy procedures, manifest surprisingly prolonged survival rates. Such cases, in which survival unexpectedly exceeds that of patients who have undergone complete surgical resections, call for a reevaluation of our current understanding of glioblastoma management and prognostication [10].

This study focused on a cohort of patients with IDH-wildtype gliomas (grade 4, WHO 2021) who only underwent biopsy, aiming to identify those who benefit from adjuvant treatments. The goal was to understand survival determinants, as such patients are rarely the focus of studies and are often mixed with those receiving multimodal treatments or having better-prognosis IDH-mutant gliomas.

## 2. Materials and Methods

### 2.1. Study Design and Subject Inclusion Criteria

We performed a retrospective analysis focusing on patients who underwent biopsy only at our institution from 2017 to 2021, diagnosed with IDH-wildtype glioblastomas as per the WHO 2021 classification. Eligibility criteria for this study were patients aged over 18 years, who had undergone a biopsy revealing an IDH-wildtype GBM and had pre-operative MRI scans. We excluded all patients with IDH mutations from the study, ensuring that only patients with IDH-wildtype tumors were analyzed. Other exclusion criteria included patients who underwent tumor resection procedures, those with a history of another intracranial tumor, patients previously treated with chemotherapy and radiotherapy, and those without available clinical registry data.

### 2.2. Data Collection Parameters

We collected demographic details such as age at diagnosis and sex. The Charlson Comorbidity Index (CCI) was employed to evaluate and quantify the presence of comorbidities in the patient population. Furthermore, we recorded the Karnofsky Performance Status (KPS) scores to measure patients’ baseline functional status, as well as a subsequent evaluation of KPS scores prior to the initiation of adjuvant therapy (dual-timepoint assessment). We also incorporated the Neurologic Assessment in Neuro-Oncology (NANO) score to evaluate neurological status. Post-operative complications within 30 days after surgery were also meticulously recorded. We investigated cognitive impairment, reviewing data from two recognized neuropsychological assessment tools: the Montreal Cognitive Assessment (MoCA) and the Mini-Mental State Examination (MMSE). For the purpose of this analysis, cognitive function was stratified into three categories based on the scores obtained from the MoCA and MMSE. Specifically, cognitive function was considered normal for scores exceeding 26 on MoCA and 27 on MMSE. Mild Cognitive Impairment was defined by MoCA scores ranging from 18 to 26 and MMSE scores between 20 and 27. Severe cognitive impairment was identified by MoCA scores below 18 and MMSE scores under 20. Patients with severe impairment of executive function or lack of cooperation on these standardized tests were classified within the severe cognitive impairment category. Patients with absence data were excluded. We also recorded the use of medications like anticoagulants, antiplatelets, antiepileptics, and corticosteroids.

### 2.3. Genetic and Epigenetic Analysis

To identify routine genetic alterations, polymerase chain reaction (PCR) tests were conducted for IDH mutations. Pyrosequencing or methylation-specific PCR (MSP) techniques determined the *MGMT* promoter’s methylation status in tumor samples.

### 2.4. Imaging Analysis

Concerning tumor volumetry, the Brainlab Elements software (version 6.0) was utilized to calculate the volume of each tumor accurately, which allowed for a standardized assessment of tumor size across the patient cohort. For evaluating contrast uptake, we adopted a classification system based on the extent of contrast enhancement observed in imaging studies. Contrast uptake was categorized into three distinct levels: none, indicating no contrast enhancement; slight, when contrast enhancement constituted less than 15% of the total tumor volume; and high, when it exceeded 15% of the total volume. Regarding the tumor’s location, we documented the primary site of each lesion based on where most of the tumor mass was located. The side of the brain affected by the tumor (left or right hemisphere) was also noted.

### 2.5. Treatment Protocols

Following surgery, patients were treated according to the following regimens: Stupp protocol, incorporating concurrent and adjuvant radiotherapy with temozolomide chemotherapy; hypofractionated radiotherapy plus temozolomide or monotherapy (chemotherapy or radiotherapy alone). When disease progression occurs despite conventional treatment, the off-label use of bevacizumab is considered. All therapeutic decisions, including the adoption of standard and alternative treatment protocols, are deliberated upon in a multidisciplinary neuro-oncology tumor board. All treatment approaches were documented carefully. In this study, the term ‘Any Adjuvant treatment’ refers to any treatment initiated post-biopsy, such as the Stupp protocol. It also includes other supportive therapies, such as bevacizumab or alternative chemotherapy regimens, but excludes treatments like tumor treating fields (TTFs), vitamins, and similar interventions.

### 2.6. Outcome Measures

This study’s main outcomes were overall survival (OS) and progression-free survival (PFS), measured from the time of diagnosis to death from any cause, or to disease progression/death, respectively. These outcomes were analyzed in relation to patient characteristics to identify significant prognostic factors.

### 2.7. Statistical Analysis

Data were analyzed using SPSS, version 29.0. Descriptive statistics are presented as means and standard deviations for continuous variables, and frequencies and percentages for categorical variables. Survival times are presented as medians and interquartile ranges. Univariate and multivariate Cox regressions were performed to assess associations with overall survival (OS) and progression-free survival (PFS). The assumptions of Cox proportional hazards were verified using log-minus-log plots, which showed parallel curves. The effect size was measured as the hazard ratio (HR) with 95% confidence intervals. Screening for entry into multivariate Cox regression was based on a *p*-value < 0.10. Kaplan–Meier analysis with stratification was used to compare adjuvant treatment groups (yes vs. no). Variables for adjustment/stratification were Karnofsky Performance Status (KPS) ≥ 70 and age ≥ 72. The decision to select the age cut-off was based on a heatmap assessment. Logistic regression was used to evaluate the association between OS and any adjuvant treatment, adjusted for KPS ≥ 70 or age ≥ 72.

### 2.8. Ethical Considerations

This research adhered to the ethical guidelines established by our institutional review board, in alignment with the principles set forth in the 1964 Helsinki declaration and its subsequent amendments. This study’s protocol was approved by the ethics committee under the project identification number 258/2024.

## 3. Results

The pre-operative demographics and clinical features of the study cohort are summarized in Table 1. A total of 99 patients diagnosed with glioblastoma were included, with a male predominance (67.7%). The mean age at diagnosis was approximately 65.5 years, with a standard deviation (SD) of 10.9 years.

The reasoning behind the use of biopsy as a diagnostic tool in these patients was mostly the location of the tumor in eloquent areas of the brain not deemed surgically accessible or safe (46.5%), as well as the presence of multifocal lesions (45.5%), with a minority of patients performing biopsy due to the need for differential diagnosis (6.1% of the cases). An examination of the post-operative complications following biopsy reveals that a significant majority of the patient cohort (89.9%) did not experience any complications. The most common post-operative complications reported were hemorrhage and neurologic deficit, totaling 10.1% of patients.

The cohort’s comorbidity burden, assessed by the CCI, averaged 2.7 (SD 1.5). Pre-operative functional status, measured by the KPS score, was relatively preserved with a mean score of 72.2 (SD 17.5). A decline was observed in the KPS scores after surgery, with a post-operative mean of 65.7 (SD 19.8). Neurological functionality, as measured by the NANO score, had a mean score of 3.1 (SD 1.9). Cognitive function varied within the patient group, with 37.4% presenting with normal cognitive function, 47.5% with mild cognitive impairment, and 15.2% with severe cognitive impairment.

The medication profile of the cohort included a small proportion of patients on anticoagulants (6.1%) and antiplatelets (12.1%), with the vast majority receiving corticosteroids (99.0%). Tumors predominantly presented as a focal lesion in 52.5% of cases, while the remainder had multifocal disease. *MGMT* methylation status was positive in 13.1% of the cases. The mean tumor volume was 23.5 cm^3^ (SD 20.2 cm^3^). In terms of tumor appearance, most cases (79.8%) had a cystic component, with a high proportion showing significant contrast enhancement (≥15% contrast uptake) in imaging studies. A minority displayed homogeneous (7.1%) or infiltrative (13.1%) features. Regarding tumor location, the majority were found in the intrinsic/midline structure of the brain (49.5%), with other locations including the frontal (18.2%), parietal (13.1%), temporal (13.1%), occipital (5.1%), and cerebellar (1.0%) regions. Tumors were almost evenly distributed between the left (46.5%) and right (36.4%) hemispheres, with a smaller number diffusely spread (17.2%).

In this study, only 8 of the 99 patients survived beyond 24 months. Among these, the median PFS was approximately 17.12 months. The mean age at diagnosis was 59.06 years, with a predominance of male patients (75%). The average CCI was 1.875. The cohort revealed a high functional status, with a KPS score of 86, maintained both pre-operatively and post-operatively before the adjuvant therapy. The median NANO score was 1.375. A significant proportion of the patients (75%) received treatment (Stupp protocol), and bevacizumab was administered to 50% of the patients. Methylation of the *MGMT* promoter was observed in only 25% of these cases.

### 3.1. Treatment Regimens Administered Post-Biopsy

Following biopsy, the subsequent treatment approaches adopted for the cohort are described in Table 2. A subset of patients (24.2%) did not receive any adjuvant treatment post-surgery, while most patients (75.8%) underwent some form of adjuvant therapy. One-third of the patients (33.3%) were treated according to the Stupp protocol. Hypofractionated radiotherapy plus TMZ was done in 37.4% of patients. Bevacizumab was administered to 13.1% of the patients.

Table 3 and Table 4 show univariate and multivariate results for associations with PFS and OS. Median PFS was 3.6 months with interquartile range (IQR) of 1.8 to 5.9, minimum of 4 days, and maximum of 52 months. Median OS was 6.0 months with interquartile range of 3.6 to 10.7, minimum of 4 days, and maximum of 52 months.

### 3.2. Uni- and Multivariate Associations with PFS

In the univariate analysis, significant factors associated with PFS included age at diagnosis (HR = 1.03, *p* = 0.006), CCI (HR = 1.23, *p* = 0.003), pre-surgery KPS (HR = 0.98, *p* = 0.001), and pre-adjuvant KPS (HR = 0.98, *p* < 0.001) [Table 3]. The presence of mild cognitive impairment (HR = 1.91, *p* = 0.005) and severe cognitive impairment (HR = 2.18, *p* = 0.014) was also significantly associated with worse PFS, as well as multifocality (HR = 1.57, *p* = 0.032). The Stupp protocol (HR = 0.45, *p* < 0.001) and Bevacizumab (HR = 0.52, *p* = 0.029*) appeared beneficial. The employment of adjuvant treatment showed a strong association with improved PFS (HR = 0.33, *p* < 0.001). There was a notable difference in outcomes depending on whether there was uptake of the contrast agent or not. Both the presence (HR 3.99, *p* = 0.021) and absence (HR 0.7, *p* = 0.014) of contrast uptake appeared related to PFS scores in univariate analysis.

Multivariate analysis maintained significance for pre-adjuvant KPS (HR = 0.97, *p* = 0.009) and the presence of mild cognitive impairment (HR = 2.22, *p* = 0.014). The absence of contrast uptake remained significantly associated with higher PFS scores (HR = 0.68, *p* = 0.040).

### 3.3. Uni- and Multivariate Associations with OS

Univariate analysis highlighted age (HR = 1.03, *p* = 0.004), CCI (HR = 1.21, *p* = 0.008), pre-surgery KPS (HR = 0.97, *p* < 0.001), and pre-adjuvant KPS (HR = 0.98, *p* < 0.001) as significantly associated with OS [Table 4]. Severe cognitive impairment (HR = 2.28, *p* = 0.009) and multifocal status remained negative predictors of OS (HR = 1.60, *p* = 0.023). Treatment with the Stupp protocol (HR = 0.41, *p* < 0.001) and bevacizumab (HR = 0.34, *p* = 0.001) correlated with better OS. The implementation of any adjuvant treatment was highly beneficial (HR = 0.14, *p* < 0.001). The absence of contrast uptake also revealed a strong association with OS (HR = 4.28, *p* = 0.030).

Multivariate analysis highlighted the Stupp protocol (HR = 0.66, *p* = 0.248) and bevacizumab (HR = 0.27, *p* = 0.002), with the latter remaining significant. Adjuvant treatment showed a strong multivariate association (HR = 0.21, *p* < 0.001). The absence of contrast uptake maintained a strong significant association with OS in multivariate analysis (HR = 0.7, *p* = 0.015).

We also studied the association of any adjuvant treatment with survival outcome, considering 3 months as the threshold. Among the study participants, 77 (77.8%) patients survived beyond the 3-month threshold, with this higher proportion of survival significantly associated with the administration of any adjuvant treatment (*p* < 0.001). Out of the 22 patients that survived less than 3 months, 8 (36.4%) were treated with any adjuvant treatment. Within the 77 patients that survived longer than 3 months, 15 (19%) had KPS < 70. Within the 22 patients that did not survive after 3 months, 11 (50%) had KPS < 70. The proportion of patients among survivors longer than 3 months with KPS ≥ 70 was 90.3%, and for KPS < 70 it was 73.3%.

**Figure 1:**
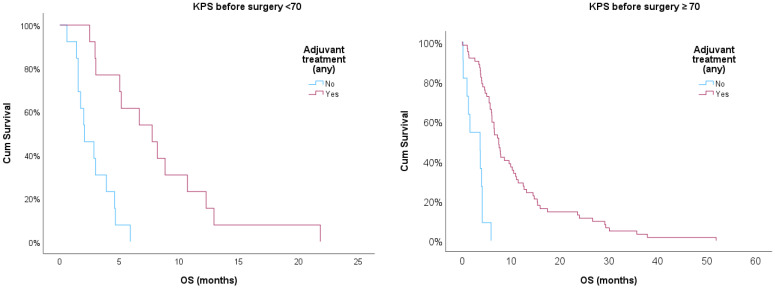
Kaplan–Meier survival curves for treated and non-treated patients with any adjuvant therapy stratified for KPS ≥ 70.

Additionally, we used Kaplan–Meier curves to compare OS for patients treated or non-treated with any adjuvant therapy, also adjusting for KPS ≥ 70. The log-rank test showed statistical significance for treated vs. non-treated patients (*p* < 0.001) after adjusting for KPS ≥ 70. Treated patients showed better prognosis in both strata, but those with KPS ≥ 70 had a better prognosis when compared with patients with KPS < 70 (*p* < 0.05). [Figure 1]

**Figure 2:**
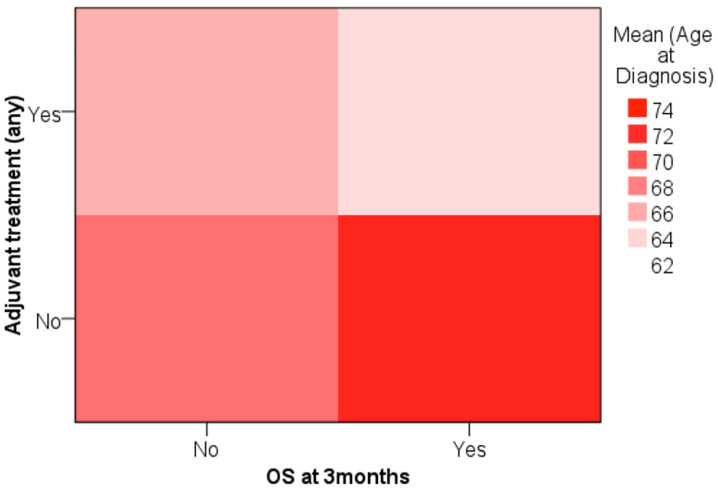
Heat map analysis of age at diagnosis in relation to adjuvant treatment administration and overall survival at 3 months. The color intensity represents the mean age at diagnosis, with darker shades indicating an older population.

We analyzed age data, attempting to define a cut-off for treatment decision considering OS at 3 months. The heat map shows that younger patients that survived longer than 3 months were more likely to have been treated with any adjuvant treatment, while older patients that survived were less likely to have received adjuvant treatment (Figure 2).

Based on the previous analysis, a cut-off was established for age ≥ 72 (n = 29, 29.3%). Within the 77 patients that survived after 3 months, 55 had age < 72 and 22 had had age ≥ 72. Within the 22 patients that did not survive after 3 months, 15 had age < 72 and 7 had age ≥ 72. The proportion of treated patients for survivors > 3 months with age ≥ 72 was 72.7%, and for age < 72 it was 92.7%. The proportion of treated patients for non-survivors > 3 months with age ≥ 72 was 14.3%, and for age < 72 it was 46.7%.

**Figure 3:**
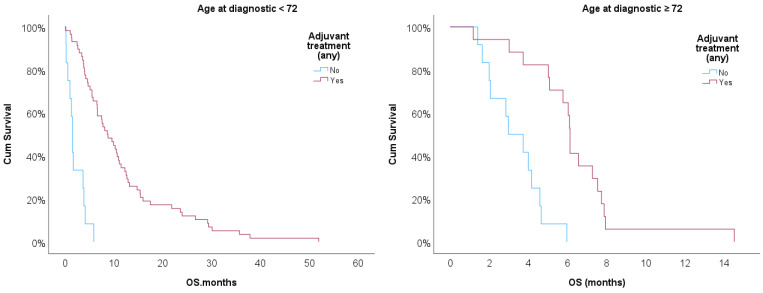
Kaplan–Meier survival curves for treated and non-treated patients with any adjuvant therapy stratified for age ≥ 72.

Kaplan–Meier curves were then implemented to compare OS for patients treated or non-treated with any adjuvant therapy adjusted for age ≥ 72 (Figure 3). The log-rank test showed statistical significance for treated vs. non-treated patients (*p* < 0.001) after adjusting for age ≥ 72. Treated patients showed better prognostic in both strata, but age ≥ 72 had a worse prognosis when compared with patients with KPS < 70 (*p* < 0.05).

The age effect was studied as a covariate of treatment effect in OS ≥ 3 months. Univariate logistic regression showed an effect of OR = 11.73 (*p* < 0.001), 95% CI = [3.93–35.00] for treatment, suggesting that treatment is associated with increased likelihood of surviving after 3 months. After adjusting for age, treatment effect on survival after 3 months was maintained (OR = 12.17, *p* < 0.001, 95% CI = [3.84–38.54]).

**Figure 4:**
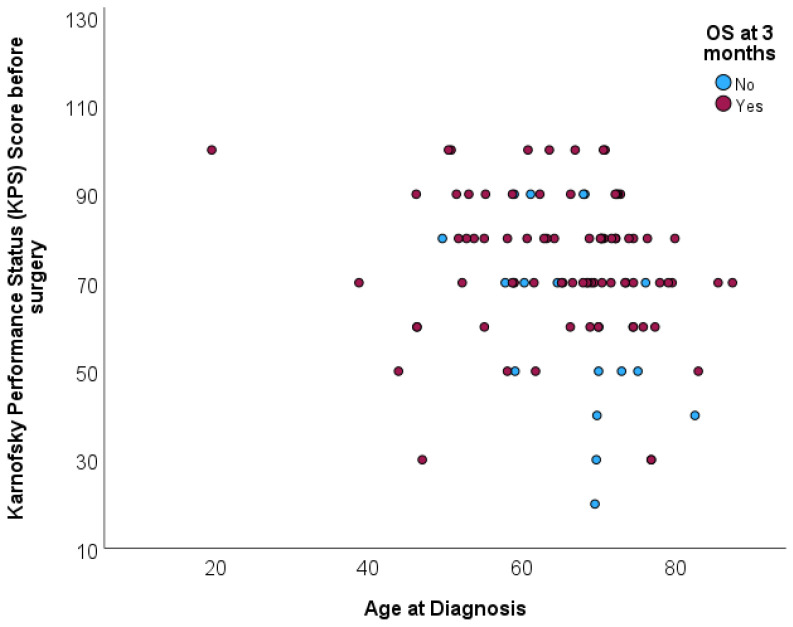
The scatterplot illustrates the distribution of patients’ KPS scores before surgery against their ages at diagnosis, with the survival outcome at 3 months post-surgery differentiated by color.

The distribution of age at diagnosis was not associated with overall survival at 3 months (*p* = 0.296). The mean age for patients deceased before completing 3 months of follow-up was 67.6 (SD = 7.9), and the mean age for patients deceased after completing 3 months of follow-up was 64.8 (SD = 11.6). The scatterplot shows the age distribution of patients according to survival beyond 3 months, without any specific pattern (Figure 4).

In conclusion, our data show that age per se does not determine the outcome of patients with IDH-wildtype glioblastoma undergoing biopsy.

## 4. Discussion

This study aimed to isolate a specific cohort of patients (IDH-wildtype) defined as grade 4 according to the WHO 2021 criteria submitted to biopsy. These patients often escape the follow-up of a neurosurgeon. It is important to understand which patients actually undergo adjuvant treatment and benefit from it in order to assess whether the decisions made in this difficult subgroup of patients are appropriate [1]. On the other hand, some of these patients rarely exhibit unexpected survival rates, and trying to identify them is also important. An attempt was made to characterize this cohort to better understand their unique survival determinants. Studies specifically focusing on IDH-WT gliomas that have experienced only biopsy are scarce and are often incorporated into research involving multimodal treatments, including surgery [8]. Additionally, such studies frequently include patients with IDH mutations who typically exhibit a better prognosis and higher rates of *MGMT* promoter methylation [2,11,12].

The observations previously outlined, coupled with the defined inclusion and exclusion criteria, partially explain the findings of our investigation regarding the diminished PFS of 3 months and OS of 6 months within our cohort. These outcomes invariably fall below the standards described in the recent literature for survival following glioma biopsies (OS~8 months) [12,13,14,15]. Considering this, it is evident that patients initially subjected to biopsy and lacking IDH mutation exhibit a prognosis that is comparatively less favorable than their counterparts.

Even though patients undergoing biopsy generally exhibit a worse prognosis compared to the overall population, there exists a very specific group of patients in our study—approximately 8%—who demonstrated a survival rate of more than 24 months. Interestingly, these patients also had a considerably high PFS of 17.12 months. These individuals were younger, had no comorbidities, were in good neurological condition, and received first-line adjuvant treatment. Although our molecular study was limited, we observed that only 25% of these patients exhibited *MGMT* promoter methylation. It has been noted that a very long survival can occur even in cases where only a biopsy was performed, without subsequent surgical resection. This finding is intriguing and somewhat paradoxical given the generally accepted belief that more aggressive surgical interventions typically correlate with better outcomes. According to the systematic review by Tomasz Tykocki and Mohamed Eltayeb, “Ten-year survival in glioblastoma. A systematic review,” approximately 5.56% of long-term survivors (9 out of 162 cases reviewed) underwent only a biopsy without subsequent surgical resection. In this study, a longer progression-free interval strongly correlates with better OS, and younger age at diagnosis has been consistently associated with increased chances of reaching ten-year survival [16].

Different studies show that patients who do not experience relapse often have tumors that lack methylation of the *MGMT* promoter. This is particularly interesting, since the presence of *MGMT* promoter methylation has traditionally been associated with better responses to alkylating agent therapy and longer survival times [2,12]. This observation suggests the presence of one or more currently unrecognized, but important, molecular or other predictors of long-term survival [11]. It is important to highlight that alterations in MYB, MN1, and the MAPK pathways typically correlate with improved survival outcomes. In contrast, the presence of mutations such as *CDKN2A*, *TERT* promoter mutations, EGFR amplifications, H3F3A alterations, and concurrent gain of chromosome 7 and loss of chromosome 10 tend to be associated with poorer survival. These factors may significantly influence prognosis and were not included in our analysis [14].

Another interesting point to reflect on was that 24.2% of patients (n = 25) did not receive adjuvant treatment. Interestingly, this aligns with the literature, which report a similar rate of 24.8% [17]. If we consider this, it becomes evident that one-quarter of patients did not get any benefit from the treatment, suggesting that the risk–benefit ratio of the procedure should be better evaluated. For this reason, liquid biopsies, an evolving alternative to conventional brain tumor biopsy, offer a minimally invasive method for early detection, monitoring, and treatment modification. These biopsies include the analysis of circulating tumor DNA (ctDNA) or RNA (ctRNA) in various biofluids, including blood and cerebrospinal fluid (CSF). This method considerably decreases the risks and discomfort associated with surgical tissue sampling, while enabling healthcare professionals to track tumor evolution and response to treatment over time, and can be an interesting area of focus in the future [18]. Furthermore, not all patients are discussed with the oncology team to assess their capability to start adjuvant treatments, and a multidisciplinary decision pre-biopsy may be the answer to decrease this percentage. Another factor that may explain these findings is the decrease in KPS from the pre-biopsy period to the first oncology consultation one-month post-procedure, where patients begin treatment. The KPS dropped slightly, with a mean score of 72.2 pre-operatively, declining to a mean of 65.7 post-operatively. The univariate and multivariate analyses suggest that a higher pre-surgery KPS is associated with a lower hazard ratio for both progression-free survival and overall survival, indicating better outcomes. Specifically, each point increase in KPS was associated with a 2% reduction in the risk of progression or death before adjuvant therapy. It should also be noted that only 33% of patients received the first-line Stupp protocol. Another 33% received hypofractionation, which is typically administered in our department to older patients with a lower capacity to tolerate increased doses of radiation.

The univariate and multivariate analyses suggest that higher pre-surgery KPS is associated with a lower hazard ratio for both PFS and OS, indicating better outcomes. This finding aligns with existing studies highlighting the importance of KPS [16,17,19]. Our study further confirms the importance of this parameter as an independent predictor of survival. However, it should not be used as the single decisive factor. Although KPS is an important predictor of survival, patients with KPS < 70 who underwent biopsy and received adjuvant treatment showed better survival compared to those who did not receive any treatment. It is important to analyze the data carefully, as KPS refers to levels of functionality. For instance, a patient with good cognitive status, no comorbidities, and young age, but who presents with paresis that prevents independence, can still benefit from treatments. We also highlight the relationship between cognitive function and clinical outcomes. Cognitive impairment, particularly mild and severe levels, was associated with worse PFS, with hazard ratios indicating almost twice the risk of progression in patients with mild impairment, and even higher in those with severe impairment. This association emphasizes the importance of neurocognitive assessments [19]. Finally, the absence of contrast uptake on imaging manifests a significant association with OS in multivariate analysis (*p* = 0.015). This parameter acquired considerable importance in therapeutic decision-making, nearly as much as KPS. Often, it does not appear as an important predictor in the literature and should be valued accordingly.

The management of glioblastomas among the elderly mirrors strategies applied to younger adults, emphasizing the safety and benefits of maximum resections. The rationale behind favoring biopsy over aggressive resection in older patients has historically been supported in the supposed poor prognosis, increased surgical risks, and unclear survival benefits. However, this perspective may overlook the significant advantages of maximum resections, challenging the premise that conservative approaches are invariably preferable in the elderly. Additional evidence suggests that the diminished prognosis for HGGs in older individuals might be more attributable to differences in tumor biology than age alone [20]. The debate over the risks of surgical resection versus biopsy and adjuvant therapy in older patients is supported by the appreciation that elderly individuals may have a reduced capacity to tolerate major surgeries and treatments due to comorbidities and diminished physiological reserves [20,21]. We attempted to establish a potential age-related cut-off for treatment decisions. The results indicated that while younger patients (age < 72) were more likely to receive adjuvant therapy, age did not significantly impact the effectiveness of the treatment in terms of survival at the 3-month mark. In fact, treatment was a strong predictor of 3-month survival irrespective of age, with an odds ratio of 12.17 and no observed effect of age on survival, suggesting that the benefits of adjuvant therapy are not age-dependent. Moreover, the lack of a significant age effect on the efficacy of adjuvant therapy suggests that older patients should not be precluded from such treatment based solely on biologic age [22].

Our study did not focus on the importance of quality of life (QoL) assessments in patients with glioblastomas. QoL is an undervalued component of patient care, particularly in the context of glioblastoma, where survival rates are notably low, and all efforts are done to increase survival. We cannot disregard the increases in fatigue post-surgery and treatments, particularly pain during chemotherapy. A marked decrease in QoL is clear, specifically after radiotherapy and in the initial months of chemotherapy. Deciding which patients are likely to gain more from palliative care can be a valuable aspect of their management [23]. Another important limitation is that many patients were referred to palliative care without undergoing biopsies and, as such, were not included in this study. Bevacizumab’s impact was noteworthy in univariate analysis (*p* = 0.029), and it appeared as an independent factor upon multivariate analysis (*p* = 0.002). Only patients in good general condition and those who had a good response to first-line treatment were referred for this off-label therapy, and therefore these results should be evaluated with caution. Although we meticulously documented all treatment approaches, the choice of treatment was not randomized and was influenced by patient status, tumor biology, and multidisciplinary team discussions that can vary between different regions and protocols. Additionally, not all patients underwent *MGMT* promoter methylation testing, and many other molecular alterations were not assessed.

## 5. Conclusions

Our study exhibits an extensive investigation of the prognostic factors influencing survival outcomes in a cohort of patients diagnosed with IDH-wildtype glioma submitted to biopsy. This study highlights the importance of KPS both before and after surgery and any form of adjuvant treatment in determining patient outcomes. A decline in KPS post-operatively was indicative of poorer prognosis. We also clarified the adverse impact of cognitive impairment on PFS and OS for patients with mild cognitive deficits, and even higher for those severely impaired. Contrary to traditional fears regarding aggressive treatments in older patients, our data did not show a significant age-related impact on the three-month survival rate after adjuvant therapy in patients able to comply with treatment. While the volumetry, location, and hemisphere dominance of gliomas did not appear as significant predictors, lack of contrast uptake in imaging studies did, and it was associated with a notably poorer PFS and OS, as an independent variable. Finally, a critical observation from our data is the clear survival benefit associated with adjuvant therapies post-biopsy, irrespective of their pre-operative clinical status.

## Figures and Tables

**Table 1:** Pre-operative characteristics of patients with glioblastoma.

	Descriptive Statistic
Gender	
Female	32 (32.3%)
Male	67 (67.7%)
Age at Diagnosis	65.5 (10.9)
CCI	2.7 (1.5)
KPS Score Before Surgery	72.2 (17.5)
KPS Before Adjuvant (After Surgery)	65.7 (19.8)
NANO	3.1 (1.9)
Cognitive Function	
Normal	37 (37.4%)
Mild Cognitive Impairment	47 (47.5%)
Severe Cognitive Impairment	15 (15.2%)
Medication	
Anticoagulants	6 (6.1%)
Antiplatelets	12 (12.1%)
Corticosteroids	98 (99.0%)
Antiepileptics	20 (20,2%)
Focality	
Focal	52 (52.5%)
Multifocal	47 (47.5%)
Tumor Features	
*MGMT* Methylation	13 (13.1%)
*MGMT* Methylation Not Tested	50 (51%)
Tumor Volumetry	23.5 (20.2)
Appearance	
Homogeneous	7 (7.1%)
Cystic	79 (79.8%)
Infiltrative	13 (13.1%)
≥15% of Contrast Uptake	86 (86.9%)
<15% of Contrast Uptake	3 (3.0%)
None	10 (10.1%)
Location	
Cerebellum	1 (1.0%)
Frontal	18 (18.2%)
Intrinsic/Midline	49 (49.5%)
Occipital	5 (5.1%)
Parietal	13 (13.1%)
Temporal	13 (13.1%)
Side	
Left	46 (46.5%)
Diffusely spread	17 (17.2%)
Right	36 (36.4%)

Note: Results presented as means (standard deviations) for continuous variables and frequencies (percentages) for categorical variables. Volumetry is presented as cm^3^. Abbreviations: CCI—Charlson Comorbidity Index, KPS—Karnofsky Performance Status, NANO—Neurologic Assessment in Neuro-Oncology, *MGMT*—O^6^-Methylguanine-DNA Methyltransferase.

**Table 2:** Treatment.

	Descriptive Statistic
**No Adjuvant Treatment**	24 (24.2%)
**Adjuvant Treatment (any)**	75 (75.8%)
Stupp Protocol	33 (33.3%)
Hypofractionation	37 (37.4%)
Bevacizumab	13 (13.1%)

Note: The table enumerates the proportions and frequencies of patients receiving each treatment modality.

**Table 3:** Uni- and multivariate associations with PFS.

	Univariate			Multivariate		
	HR	*p*-Value	95% CI	HR	*p*-Value	95% CI
Gender	1.24	0.325	0.81–1.90	-	-	-
Age at Diagnosis	1.03	0.006 **	1.01–1.05	1.00	0.941	0.96–1.04
CCI	1.23	0.003 *	1.07–1.42	1.06	0.744	0.76–1.46
KPS Score Before Surgery	0.98	0.001 **	0.97–0.99	1.04	0.062	1.00–1.08
KPS Score Before Adjuvant	0.98	<0.001 ***	0.96–0.99	0.97	0.009 **	0.94–0.99
NANO	1.18	0.002 **	1.06–1.31	1.07	0.541	0.86–1.34
Cognitive Function (REF = normal)						
Mild Cognitive Impairment	1.91	0.005 **	1.21–2.99	2.22	0.014 *	1.17–4.21
Severe Cognitive Impairment	2.18	0.014 *	1.17–4.05	1.42	0.410	0.62–3.27
Medication Use: Anticoagulants	1.27	0.576	0.55–2.94	-	-	-
Medication Use: Antiplatelets	0.91	0.753	0.49–1.67	-	-	-
Medication Use: Corticosteroids	4.78	0.128	0.64–35.78	-	-	-
Focal (REF) vs. Multifocal	1.57	0.032 *	1.04–2.36	1.40	0.155	0.88–2.21
*MGMT* Methylation	1.26	0.438	0.70–2.27	-	-	-
Treatment: Stupp Protocol	0.45	<0.001 ***	0.29–0.69	0.73	0.370	0.37–1.45
Treatment: Hypofractionation	0.98	0.911	0.65–1.47	-	-	-
Treatment: Bevacizumab	0.52	0.029 *	0.29–0.93	0.53	0.125	0.23–1.19
Adjuvant Treatment (any)	0.33	<0.001 ***	0.20–0.54	0.99	0.987	0.45–2.21
Tumor Volumetry	1.01	0.062 ^‡^	1.00–1.02	1.00	0.674	0.98–1.01
Tumor Features (REF = Homogeneous)						
Cystic	1.32	0.484	0.60–2.91	-	-	-
Infiltrative	0.85	0.727	0.33–2.16	-	-	-
Contrast Uptake (REF = Slight)						
No contrast uptake	0.70	0.014 *	0.50–0.95	0.68	0.040 *	0.45–0.98
≥15% of contrast uptake	3.99	0.021 *	1.23–12.95	2.76	0.134	0.73–10.41
Side (REF = Midline)						
Left	0.81	0.493	0.47–1.44	-	-	-
Right	1.01	0.984	0.56–1.80	-	-	-

^‡^ *p* < 0.10; * *p* < 0.05; ** *p* < 0.01; *** *p* < 0.001; HR: hazard ratio; 95% CI: 95% confidence interval.

**Table 4:** Uni- and multivariate associations with OS.

	Univariate			Multivariate		
	HR	*p*-Value	95% CI	HR	*p*-Value	95% CI
Gender	1.22	0.372	0.79–1.87	-	-	-
Age at Diagnosis	1.03	0.004 **	1.01–1.05	0.99	0.668	0.95–1.04
CCI	1.21	0.008 **	1.05–1.39	1.06	0.722	0.76–1.48
KPS Score Before Surgery	0.97	<0.001 ***	0.96–0.99	1.03	0.090	0.99–1.08
KPS Score Before Adjuvant	0.98	<0.001 ***	0.96–0.99	0.99	0.363	0.97–1.01
NANO	1.21	<0.001 ***	1.09–1.34	1.16	0.207	0.92–1.45
Cognitive Function (REF = normal)						
Mild Cognitive Impairment	1.55	0.051 ^‡^	1.00–2.40	1.61	0.156	0.83–3.10
Severe Cognitive Impairment	2.28	0.009 **	1.23–4.24	1.41	0.458	0.57–3.48
Medication Use: Anticoagulants	1.85	0.150	0.80–4.29	-	-	-
Medication Use: Antiplatelets	1.09	0.780	0.59–2.00	-	-	-
Medication Use: Corticosteroids	2.66	0.332	0.37–19.25	-	-	-
Focal (REF) vs. Multifocal	1.60	0.023 *	1.07–2.41	1.49	0.117	0.91–2.44
*MGMT* Methylation	0.96	0.888	0.53–1.73	-	-	-
Treatment: Stupp Protocol	0.41	<0.001 ***	0.27–0.64	0.66	0.248	0.33–1.33
Treatment: Hypofractionation	0.94	0.785	0.63–1.43			
Treatment: Bevacizumab	0.34	0.001 **	0.19–0.63	0.27	0.002 **	0.12–0.61
Adjuvant Treatment (any)	0.14	<0.001 ***	0.08–0.25	0.21	<0.001 ***	0.09–0.49
Tumor Volumetry	1.01	0.123	1.00–1.02	-	-	-
Tumor Features (REF = Homogeneous)						
Cystic	1.14	0.743	0.52–2.48	-	-	-
Infiltrative	0.88	0.782	0.35–2.21	-	-	-
Contrast Uptake (REF = Little)						
No	0.65	0.030 *	0.45–0.93	0.70	0.015 *	0.50–0.98
Yes	2.97	0.068	0.92–9.58	4.02	0.062	0.93–17.35
Side (REF = Midline)						
Left	0.59	0.070 ^‡^	0.33–1.05	0.58	0.577	0.28–1.18
Right	0.78	0.407	0.44–1.40	0.69	0.691	0.33–1.45

^‡^ *p* < 0.10; * *p* < 0.05; ** *p* < 0.01; *** *p* < 0.001; HR: hazard ratio; 95% CI: 95% confidence interval.

## Data Availability

The original contributions presented in the study are included in the article, further inquiries can be directed to the corresponding author.
